# Zing-Yang Kuo and behavior epigenesis based on animal experiments

**DOI:** 10.1007/s13238-018-0516-9

**Published:** 2018-03-05

**Authors:** Yanyan Qian, Wei Chen, Benyu Guo

**Affiliations:** 1 School of psychology, Nanjing Normal University, Nanjing, 210097 China; 2 Department of psychology, Shaoxing University, Shaoxing, 312000 China; 3 Department of psychology, The Education University of Hong Kong, Hongkong, China

Zing-Yang Kuo (1898–1970) (Fig. 1), styled Taofu, was a world-renowned Chinese psychologist. He was one of the most extreme precursors of the behaviorism school and the most radical behaviorist in the history of behavioristic psychology. His main scope of research focused on animal and comparative psychology. Animal experimentation was his favorite research method, and he studied many different species of animals. The aim of Kuo’s research was to determine the origin of the formation of behavior, and in 1967, he constructed a theory for this formation called behavior epigenesis. Kuo was the only Chinese psychologist profiled in *The First Century of Experimental Psychology*. In addition, the *Journal of Comparative and Physiological Psychology*, which usually only publishes scientific experiments, provided a few pages to discuss the life of Kuo and gave this high appraisal: “During his turbulent career he stood at the center of several important metatheoretical controversies and made unique investigative contributions to the study of behavior and the nervous system, particularly from the standpoint of developmental analysis (Gottlieb, 1972).”

**Figure 1:**
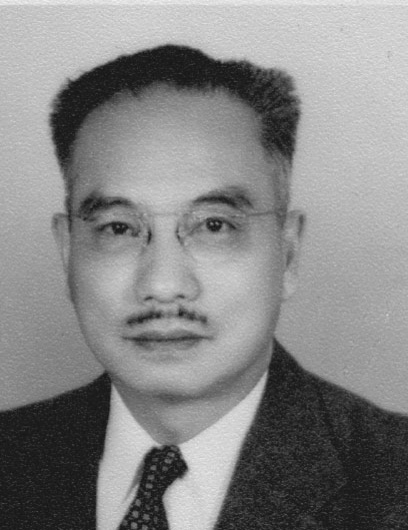
Zing-Yang Kuo in 1955

In 1898, Kuo was born to a merchant family in Tongyu Village, Chaoyang County, Guangdong Province, China. He entered Fudan University in 1916 and left two years later to pursue advanced studies in the United States, where he enrolled in the University of California at Berkeley. After some vacillation, he settled on psychology as his major. In 1918, he met his supervisor, Edward Chace Tolman, a flagman in neo-behaviorism, who began at the University of California the same year. During the years of 1921 and 1922, he worked as an assistant at Berkeley, completed his studies and met the requirements for a Doctor of Philosophy in 1923. However, Kuo did not agree with the amendments put forward by school authorities on his doctoral thesis and instead persisted in his academic viewpoints. He thus gave up the chance to earn his Ph.D. and returned to China before his oral thesis defense (Kuo, 1940). He would not earn a doctoral degree until 1936.

In 1923, Kuo returned to his homeland and took a series of positions at Fudan University between 1923 and 1927. He was a professor in 1923, acted as vice-president and exercised the power and duties of acting-president in 1924, and served as acting-president until 1926. During this time, he set up a department of psychology in 1924 and raised money from clansmen to establish Sub Bin hospital (Fig. 2)—the third largest Institute of Psychology in size according to Shun Pao (Hu, 1995) and the only Institute of Psychology in China at that time.

**Figure 2:**
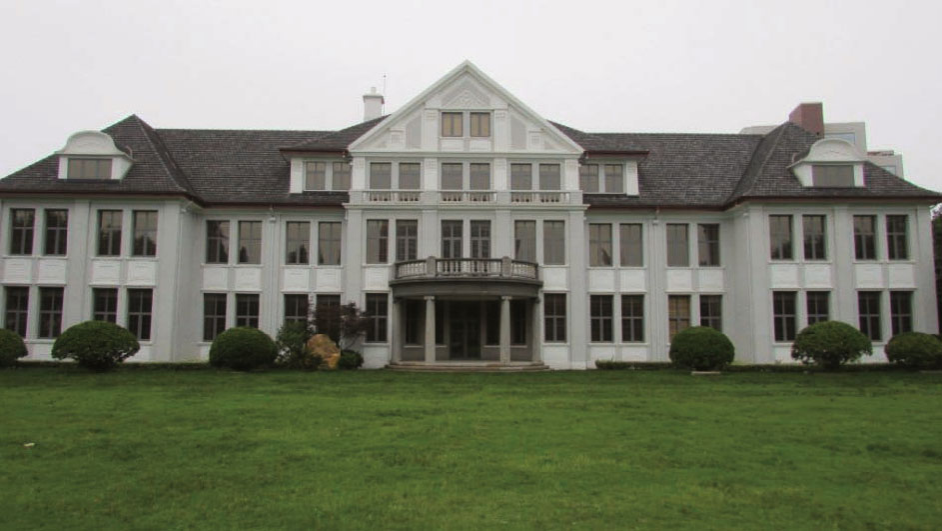
Sub Bin hospital

The following year, Kuo held a teaching post at Nanjing Central University, where he founded the Psychology Institution in Academia Sinica while acting as the head of this research institute. From April 1933 to February 1936, he held a position at Zhejiang University, where he imparted knowledge and educated people. He occupied the role of schoolmaster, launched the department of psychology, and was elected to the First Research Council of Academia Sinica in 1935. He also took part in the construction of a psychology association: Kuo and eight other psychologists (Kuo Itzen, AI Joseph Wei, Hsiao Hsiao-Hung etc.) launched the Chinese Psychological Association and held a preliminary meeting in Shanghai during the summer session of 1931.

On December 10, 1935, a student movement erupted in Zhejiang, led by Zhejiang University students, that opposed by Kuo. This conflict caused Kuo’s deposition which announced in 257th regular meeting of Executive Yuan in February, 1936. This further led to a change in his research methods and prospects. From then on, Kuo became a wandering scientist without a nation to call home. From 1936 to 1946, he gave lectures on psychobiology at Berkeley, performed research at the Osborn Zoological Laboratory of Yale University (1937–1938) and the Carnegie Institution of Washington (1938–1939), and went on a lecture circuit of several universities (1941–1943).

In 1946, Kuo became a member of the Board of Trustees of the University of Hong Kong. During the next twelve years, he occupied himself with studies of human behavior, including a social psychological analysis of the Chinese national character (Gottlieb, 1972). In 1963, Kuo stayed in America for months to popularize his scientific ideas, including making speeches at the meeting of the International Congress of Psychology and the International Congress of Zoology in August in Washington, DC (Kuo, 1967). That same year, Kuo returned to Hong Kong, where he passed away on August 14, 1970.

Kuo’s research process can be divided into three stages: theoretical assumption, animal experimentation and theory formation (Fig. 3) (Gottlieb, 1972). In Kuo’s opinion, psychology should be an empirical science. He believed that instincts led away from empirical science: “Whether all the opponents of instincts will agree or not, I believe our chief motive for denying instincts is to rescue psychology from armchair speculation. We mean to remove this stumbling-block from genetic psychology (Kuo, 1922)”. Thus, he published *Giving Up Instincts in Psychology* when he was a junior (3rd year) student to prove that “Instinct was a finished psychology” (Kuo, 1922) and became the first person to argue against the concept and classification of instinct. This paper garnered widespread academic attention in American psychology academy. Subsequently, Kuo received support from other psychologists (Allport, Ayers, Bernard, Dunlap, Faris, Josey, Kantor, etc.) who objected to the concept of instinct. In contrast, world-renowned psychologists such as William McDougall, who supported the concept of instinct, wrote articles in response to Kuo, spurring the famous anti-heredity movement.

**Figure 3:**
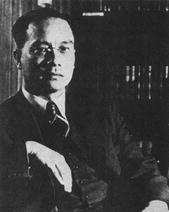
Zing-yang Kuo (1898–1970)

Kuo made considerable efforts to carry out a large number of experiments on the animal behavioral response. These experiments sought to observe and determine the rules governing animal ontogeny and development. His series of experiments can be grouped into three categories. The first pertained to the genesis of the cat’s response to the rat. These experiments showed the behavior of kittens toward rats and mice under different environmental conditions: “Our study has shown that kittens can be made to kill a rat, to love it, to hate it, to fear it or to play with it (Kuo, 1930)”. He pointed out that both ontogeny and development play important roles in the formation of the cat’s response to the rat (Kuo, 1938).

In the second vein of research, he studied the factors that determine fighting in animals. Kuo employed a large number of species, such as fighting crickets, Siamese fighting fish, Japanese grey quails, chickens, dogs, numerous species of fish, more than 30 species of birds, cats, rats, rabbits and guinea pigs, recording and classifying all fighting behavior patterns into 23 types. Experiments further revealed that nutritional, hormonal, developmental and environmental factors play an important role in the fighting behavior patterns of the Japanese grey quail (Kuo, 1960a, b, c, d), and he described the phenomenon of inter-species coexistence in fish, birds and mammals (Kuo, 1960e, f, g).

Third, he studied the behavior and physiology of the embryonic nervous system, mainly through a series of behavioral studies on chick embryos, which he systematically presented in several articles on the subject of the ontogeny of embryonic behavior in Aves (Fig. 4). He created the Kuo Observation Window, which is regarded as one of the most well-known experiments, and he provided the first record of a method for opening the egg and studying the embryo without inducing harm. Based on these studies, Kuo was recognized as a great scientist, not only by psychologists but also by biologists (Scheithauer et al., 2009). Kiessling and Anderson (2003) praised his work, saying “Kuo was the first to peer inside an egg since Aristotle did so”. Not only his research findings but also his experimental methods were taught to later generations of scholars, for example, “Gottlieb employed Kuo’s methods to begin his pioneering series of experiments on the effects of experience on ducklings’ recognition of their species’ maternal assembly call (Logan and Johnston, 2007)”.

**Figure 4:**
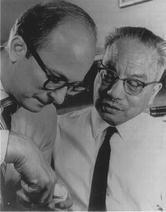
Zing-Yang Kuo and Gilbert Gottlieb in North Carolina in 1963

Over many years of the study of the development of behavior in the avian embryo, the development of the behavior of the cat in relation to the rat, and particularly the fighting behaviors of a number of species, Kuo proposed new theoretical concepts in his book, *The Dynamic of Behavior Development*, which discussed behavior epigenesis, behavior gradients and behavioral potential. Kuo defined behavior epigenesis as the idea that every animal is a result of its developmental history and it will react to a specific stimulus in a specific environmental setting. He expressed this view in a simple formula^1^:Besides, the theory of behavior gradients present information about the developmental sequence of behavior, and the theory of behavioral potential provide information of the origin and developmental sequence of behavior, i.e., the cause or causes for the appearance of certain behavior patterns at different stages of ontogeny (Kuo, 1967).

Logan and Johnston (2007) spoke highly of Kuo’s work: “Kuo was the first empirically supported statement of the necessity of transcending the separation between nature and nurture in order to understand behavior”. Kuo’s revolutionary work embodies what today we call a relational developmental systems (RDS) perspective (Overton and Lerner, 2012), and it contributed to the Kuhnian paradigm shift in developmental science (Greenberg, 2014). In a word, Zing-Yang Kuo was a special Chinese psychologist of great significance to the history of psychology and whose research represents a vast treasure of resources that could be developed and used in the future in many different fields.

## Footnotes

1 DH = developmental history, ST = special training (such as fighting, sensory discrimination, etc.), SS = special stimulus (such as light, sound, food, sex, objects, etc.), BCF = biochemical factors, and Pred.Beh. = predictable behavior. Bracket (1) deals with the animal’s past, (2) with its present, and (3) with its physiological mechanisms.
